# P05.38. Experiences and wishes from cancer children and their families regarding CAM (complementary and alternative therapies)

**DOI:** 10.1186/1472-6882-12-S1-P398

**Published:** 2012-06-12

**Authors:** L Eriksen

**Affiliations:** 1 CareCam - FCB, Association of Cancer Children in DK, Vallensbaek, Denmark

## Purpose

To illuminate children with cancer and their families’ experiences and wishes regarding CAM, and to develop a model for data collection, which may be internationally useful.

## Methods

An exploratory approach using mixed methods was done in two phases. Phase One: Focus group consisting of families of children with cancer. Exchange of experiences based on 7 questions regarding traditional / conventional and CAM treatment. Phase Two: Completion of an anonymous questionnaire, also containing 7 questions.

## Results

With a representation of 51 families of children with cancer, 26 questionnaires were returned (13 boys and 13 girls), corresponding to a 51% response. In addition, 5 forms were received, completed by adults (data analysis is currently taking place, November 2011).

## Conclusion

We know very little about which CAM treatments work and how they work. Families of children with cancer have experiences with CAM and want access to sober objective user information. They also wish to see CAM being integrated within the health services as a supplement to conventional treatment (Detailed information will be available at the ICCMR Congress May 2012).

